# Prevalence of antiemetic administration after abdominal surgery with or without a regional anesthesia under general anesthesia in a nation-wide population-based study

**DOI:** 10.1097/MD.0000000000042894

**Published:** 2025-08-22

**Authors:** Manabu Yoshimura, Takaaki Tanemoto, Hiroko Shiramoto, Mami Koga, Yasuhiro Morimoto

**Affiliations:** a Department of Anesthesiology, Ube Industries Central Hospital, Ube, Japan.

**Keywords:** abdominal surgery, epidural anesthesia, peripheral nerve block, postoperative nausea and vomiting, regional anesthesia

## Abstract

The difference in the effect of regional anesthesia (RA; peripheral nerve block or epidural anesthesia) combined with general anesthesia (GA) and that of GA alone on postoperative nausea and vomiting (PONV) remains unclear. We used a national Japanese clinical database to evaluate whether the PONV incidence differed between GA with RA and GA alone during abdominal surgery. In this retrospective nation-wide cohort study, we compared the outcomes of patients who received GA with RA with those of patients who received GA alone during abdominal surgery between April 2016 and October 2019. The primary outcome was PONV, which was defined as antiemetic use within 2 days of surgery. Covariates were used to stabilize the inverse probability of treatment weighting. Univariate and multivariate Cox proportional hazard regression analyses were performed. E-values, subgroup definition, and restricted mean survival time were used for sensitivity analyses. Among the 566,819 patients who met the eligibility criteria, 249,433 received GA–RA and 317,386 received GA alone. Overall, 148,105 patients (59.4%) in the GA–RA group and 132,819 (41.8%) in the GA-alone group developed PONV. The weighted hazard ratios of the univariable and multivariable models for PONV were 1.25 (95% CI, 1.24 to 1.26; *P* < .001) and 1.20 (95% CI, 1.19 to 1.21; *P* < .001), respectively, for GA–RA and GA alone. Sensitivity analyses confirmed the robustness. GA–RA is associated with a slight increase in PONV. Therefore, opioids may be used sparingly when combined with RA.

## 1. Introduction

Postoperative nausea and vomiting (PONV) remain a common problem in the perioperative period because anesthesiologists have yet to develop valuable countermeasures. The incidence of PONV in the general surgical population is approximately 30%.^[1]^ It is also associated with more extended hospital stays and higher healthcare costs.^[2,3]^

Currently, general anesthesia (GA) combined with regional anesthesia (RA; epidural anesthesia [EA] or peripheral nerve block [PNB]) is the standard anesthesia method for abdominal surgery in Japan. The use of PNBs under GA has increased recently and has been covered by insurance in Japan since April 2016. It is generally believed that RA decreases the incidence of PONV by decreasing postsurgical pain and opioid consumption; however, this remains uncertain. In a scoping review of 18 studies comparing PONV outcomes between RA-containing enhanced recovery programs and non-RA-containing care pathways, 5 studies found RA to have improved PONV, one found PONV to be higher in the RA group,^[4]^ and 12 found no intergroup differences (possibly due to small sample sizes).^[5]^ The extent to which PNBs reduce PONV is difficult to determine because of the high variability in the indications for and practical implementation of individual procedures.^[6]^ Thus, the authors of the PONV guidelines for gynecological and oncological surgery state that regional interventions may decrease opioid use and postoperative pain; however, this may not directly translate into a PONV advantage in all cases.^[7]^ There is no clear consensus on the effects of GA with EA either.^[8,9]^ The anesthetic regimen of GA combined with EA is often an inappropriate comparison because numerous variables and heterogeneity in anesthetic management significantly alter outcomes. Thus, there is no way to prove it with a small sample size.

In recent years, the use of big data has been increasingly applicable to solving these issues. There are limitations to this solution in prospective studies and clinical trials. In addition, the number of cases in electronic medical record information does not reach that of medical claims data. Furthermore, medical claims data is collected from multiple facilities, which increases its generalizability. Extensive and highly generalizable medical claims data could be used to investigate this issue. Therefore, we used medical claims data to detect a potential association between RA under GA and PONV incidence.

## 2. Methods

This study was prepared in accordance with the Strengthening the Reporting of Observational Studies in Epidemiology guidelines.^[10]^ Further, this retrospective cohort study was approved by the Ethics Committee of the Ube Industrial Central Hospital (approval number: 153230515; June 12, 2023), which waived the need for obtaining informed consent owing to the anonymized nature of the data.

### 2.1. Data source

Inpatient diagnosis, procedure, and combination (DPC) data were collected from the multihospital claims database provided by medical data vision Co., Tokyo, Japan (https://en.mdv.co.jp/about-mdv-database/). The database comprises data on Japanese administrative claims. It has accumulated DPC data from approximately 20 million patients admitted across 1730 hospitals, i.e., approximately 23% of the hospitals adopting the DPC system in Japan. DPC data contain the following: patient demographic information, clinical summary data (including clinical diagnosis on admission; comorbidities on admission; and complications after admission according to the International Classification of Diseases, 10^th^ revision [ICD-10], codes), claims data regarding medications and devices used, data on medical and surgical procedures defined using Japan-specific standardized codes (K codes), duration of hospitalization, and discharge status.^[11]^ Anesthesia induction records were derived from claims data.

### 2.2. Population

Patients aged 18 years or older who underwent abdominal surgery under GA between April 1, 2016, and October 31, 2019, were identified. Patients for whom the anesthesia duration was more than 1440 min, those who underwent multiple surgical procedures per admission, those who were not extubated, and those with missing values were excluded.

### 2.3. Exposure

The anesthesia induction records included information on GA and an additional charge for GA combined with PNB or with EA; patients who received GA alone were classified into the GA-alone group, and those who received GA and PNB or GA and EA were classified into the GA–RA group.

### 2.4. Outcome

The primary outcome was PONV occurrence within 2 postoperative days. PONV was defined as requiring newly prescribed antiemetic medications (droperidol, metoclopramide, hydroxyzine, and prochlorperazine). In Japan, only these drugs could be used for PONV treatment due to insurance coverage. Because 5HT3 receptor antagonists, such as granisetron and ondansetron, have been covered by insurance only since September 2021, they were not administered within the observation period. In addition, prophylactic administration of PONV drugs was not permitted by insurance.

### 2.5. Covariates

Covariates included patient characteristics, comorbidities, and hospital characteristics. Based on author-led clinical evaluation and existing literature, these covariates were chosen because they are pretreatment factors that may influence patient outcomes and the choice of the anesthesia technique. Demographic characteristics included patient age, body mass index, sex, preprocedural drug use, anesthesia induction difficulty, and smoking status. The comorbidities included serious acute preoperative complications and chronic comorbidities. Comorbidities were identified using ICD-10 codes, procedures, and medications based on Elixhauser comorbidities.^[12]^ Hospital types were classified based on patient volume. The patient emergency type was categorized as scheduled or emergency hospitalization. Surgical types were categorized on the basis of diagnosis codes.

### 2.6. Statistical analysis

The following baseline 45 variables related to both the exposure and outcomes were used to calculate the propensity scores: age, body mass index, sex, anesthesia induction difficulty, smoking status, comorbidities, emergency admission, preoperative blood transfusion, oral benzodiazepine use, corticosteroid use, hospital type, surgical type, laparoscopic surgery, and year of treatment. Logistic regression models were used in this study. c-statistics were calculated as a measure of model performance. Baseline covariates that may affect the use of RA, incidence of PONV, and balance after stabilized inverse probability of treatment weighting (sIPTW) by propensity scores were examined using absolute values of standardized differences, and differences of 10% or more were considered unbalanced. After balancing, a sIPTW Cox proportional hazard regression analysis was performed for binary variables to calculate the hazard ratios (HRs) and 95% CIs. Crude rates of the outcomes of interest were calculated at postoperative 2 days. PONV was analyzed as a time-to-event outcome using sIPTW Cox regression models to calculate the 2-day HRs. A univariate analysis was performed. Subsequent multivariable analysis included sex, age (≥ 50 year-old) duration of anesthesia (≥ 60 minutes), smoking, use of inhalation anesthesia, intraoperative and postoperative opioid use, and laparoscopic surgery, which are considered risk factors for PONV.^[7]^ The sIPTW Kaplan–Meier curves were plotted. A Schoenfeld residual test and complementary log plots were used to assess the proportional hazards assumption.

A complete case analysis was conducted for missing values. Because the present study involved all available records that met the criteria, calculations were not performed to determine the sample size. Nevertheless, the results should be interpreted with caution because of the sample size and power limitations.

All statistical analyses were performed using R 4.3 and Stata/MP version 17.0 (Table S1, Supplemental Digital Content, https://links.lww.com/MD/P214).

### 2.7. Sensitivity analysis

Sensitivity analyses were performed to test the robustness of the results. First, the E-value was calculated to investigate the strength of the association between an unmeasured confounder and the exposure–outcome relationship necessary to negate the observed results, conditional on the covariates that were adjusted for in the primary analysis.^[13]^

Second, subgroup analyses were also performed. GA with PNB and GA with EA were considered to determine the HRs calculated by subgrouping for the PONV incidence.

Third, a restricted mean survival time (RMST) analysis was performed to supplement the Cox proportional hazards model analysis. RMST refers to the mean survival time from an event over a specific time horizon.^[14]^ The absolute difference in the RMST among treatments provides an anchor for quantifying treatment effects without imposing model assumptions. In the sensitivity analyses, a weighted RMST analysis was performed using sIPTW.

Fourth, multiple imputation by chained equations was performed. Complete case analysis may introduce bias, particularly when data are not missing completely at random. Variables with missing data were imputed using multiple imputation, generating 20 imputed datasets. Appropriate predictor variables were selected for each imputation model. The same statistical model was then applied to each imputed dataset, and results were pooled using Rubin rules to calculate the mean and variance of estimates across imputations.

## 3. Results

**Figure 1.**
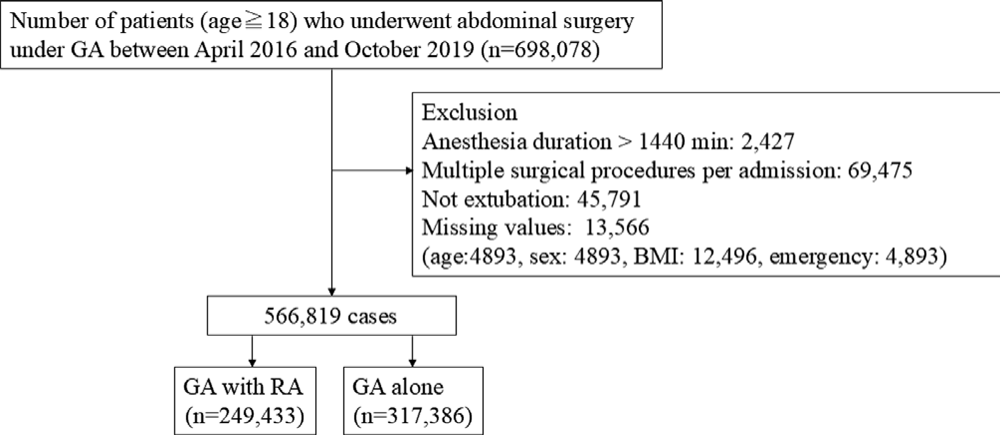
Flow chart of patient selection. GA = general anesthesia, RA = regional anesthesia (peripheral nerve block or epidural anesthesia).

We selected 698,078 patients who had undergone abdominal surgery during the study period. A total of 131,259 patients were excluded from the initial cohort for the following reasons: 2427 patients in whom the anesthesia duration was more than 1440 min, 69,475 patients who underwent multiple surgical procedures per admission, 45,791 patients who were not extubated, and 13,566 patients who had missing values. A total of 566,819 patients who met the inclusion criteria were divided into the GA–RA group (n = 249,433) and the GA-alone group (n = 317,386) (Fig. 1). Overall, 148,105 patients (59.4%) in the GA–RA group and 132,819 (41.8%) in the GA-alone group experienced PONV. Table 1 shows the baseline characteristics of the patients in each group. No significant differences in the baseline characteristics were noted between the groups after sIPTW (c-statistic: 0.72).

**Table 1:** Baseline characteristics of the patients.

		GA alone (n = 317,386)	GA with RA (n = 249,433)	Unweighted ASD	Weighted ASD
Age, n (%)	18–39	45,708 (14.4%)	27,697 (11.1%)	0.032	0.003
	40–49	42,704 (13.5%)	36,349 (14.6%)		
	50–59	37,901 (11.9%)	31,841 (12.8%)		
	60–69	64,746 (20.4%)	56,684 (22.7%)		
	70–79	77,358 (24.4%)	63,561 (25.5%)		
	80=<	48,969 (15.4%)	33,301 (13.4%)		
Sex, male, n (%)	–	164,053 (51.7%)	112,999 (45.3%)	0.128	0.004
Body mass index, kg/m^2^	<17.9	21,264 (6.7%)	19,023 (7.6%)	0.056	0.003
	18–24.9	207,598 (65.4%)	166,076 (66.6%)		
	25–29.9	71,294 (22.5%)	52,303 (21.0%)		
	30–34.9	13,622 (4.3%)	10,009 (4.0%)		
	35=<	3608 (1.1%)	2022 (0.8%)		
Preoperative corticosteroid, n (%)	–	33,222 (10.5%)	29,999 (12.0%)	0.049	0.007
Preoperative oral benzodiazepine, n (%)	–	36,918 (11.6%)	37,343 (15.0%)	0.098	0.005
Preoperative blood transfusion, n (%)	–	12,725 (4.0%)	20,202 (8.1%)	0.172	0.001
Smoking, n (%)	–	126,330 (39.8%)	99,541 (39.9%)	0.002	0.001
Anesthesia_difficult	–	26,871 (8.5%)	16,838 (6.8%)	0.065	0.004
Comorbidity on admission, n (%)					
Congestive heart failure	–	27,347 (8.6%)	19,362 (7.8%)	0.031	0.006
Cardiac arrhythmias	–	32,409 (10.2%)	25,559 (10.2%)	0.001	0.002
Valvular disease	–	22,372 (7.0%)	17,550 (7.0%)	0.001	0.001
Pulmonary circulation disease	–	2161 (0.7%)	1772 (0.7%)	0.004	0.003
Peripheral vascular disease	–	14,597 (4.6%)	8977 (3.6%)	0.05	0.006
Hypertension, uncomplicated	–	90,754 (28.6%)	74,332 (29.8%)	0.027	0.009
Hypertension, complicated	–	2361 (0.7%)	2106 (0.8%)	0.011	0.007
Paralysis	–	2083 (0.7%)	1000 (0.4%)	0.035	0.002
Other neurological disorder	–	7312 (2.3%)	4232 (1.7%)	0.043	0
Chronic pulmonary disease	–	24,911 (7.8%)	21,281 (8.5%)	0.025	0.001
Diabetes, uncomplicated	–	35,973 (11.3%)	31,128 (12.5%)	0.035	0.002
Diabetes, complicated	–	11,612 (3.7%)	8515 (3.4%)	0.013	0.003
Hypothyroidism	–	5489 (1.7%)	4289 (1.7%)	0.001	0.005
Renal failure	–	12,749 (4.0%)	7212 (2.9%)	0.062	0.012
Liver disease	–	33,917 (10.7%)	30,718 (12.3%)	0.051	0.002
Peptic ulcer disease excluding bleeding	–	45,804 (14.4%)	48,413 (19.4%)	0.133	0.002
AIDS/HIV	–	157 (0.0%)	86 (0.0%)	0.007	0.002
Lymphoma	–	2598 (0.8%)	2321 (0.9%)	0.012	0
Metastatic cancer	–	14,570 (4.6%)	26,802 (10.7%)	0.233	0.002
Solid tumor without metastasis	–	97,264 (30.6%)	142,885 (57.3%)	0.557	0.005
Reumatoid arthritis/collagen vascular disease		6109 (1.9%)	4612 (1.8%)	0.006	0
Coagulopathy	–	11,242 (3.5%)	8899 (3.6%)	0.001	0
Obesity	–	2922 (0.9%)	2183 (0.9%)	0.005	0
Weight loss	–	2552 (0.8%)	3686 (1.5%)	0.063	0
Fluid and electrolyte disorder	–	42,970 (13.5%)	37,708 (15.1%)	0.045	0.003
Blood loss anemia	–	6077 (1.9%)	8314 (3.3%)	0.089	0.002
Defficient anemia	–	44,189 (13.9%)	57,139 (22.9%)	0.233	0.008
Alcohol abuse	–	2326 (0.7%)	1983 (0.8%)	0.007	0.001
Drug abuse	–	57 (0.0%)	47 (0.0%)	0.001	0.001
Psychose	–	5215 (1.6%)	3947 (1.6%)	0.005	0.001
Depression	–	8308 (2.6%)	6334 (2.5%)	0.005	0.003
Hospital, n (%)	<199	19,621 (6.2%)	13,264 (5.3%)	0.043	0.009
	200_499	182,119 (57.4%)	141,279 (56.6%)	–	–
	500=<	115,646 (36.4%)	94,890 (38.0%)	–	–
Emergency, n (%)	–	50,768 (16.0%)	28,002 (11.2%)	0.139	0.008
Treatment, year, n (%)	2016	60,238 (19.0%)	46,923 (18.8%)	0.015	0
	2017	90,017 (28.4%)	73,095 (29.3%)		
	2018	94,522 (29.8%)	74,716 (30.0%)		
	2019	72,609 (22.9%)	54,699 (21.9%)		
Laparoscopic surgery, n (%)	–	135,178 (42.6%)	100,621 (40.3%)	0.046	0.026
Departments, n (%)					
General surgery	–	149,389 (47.1%)	136,752 (54.8%)	0.156	0.006
Gastroenterological surgery	–	56,536 (17.8%)	53,645 (21.5%)	0.093	0.009
Hepatobiliary and Pancreatic Surgery	–	79,084 (24.9%)	26,137 (10.5%)	0.385	0.005
Urology	–	527 (0.2%)	616 (0.2%)	0.018	0.001
Obstetrics and gynecology	–	12,091 (3.8%)	13,102 (5.3%)	0.069	0.007
Gynecology	–	18,272 (5.8%)	17,870 (7.2%)	0.057	0.001
Obstetrics	–	1487 (0.5%)	1311 (0.5%)	0.008	0.007

ASD = absolute standardized difference, EA = epidural anesthesia, GA = general anesthesia, PNB = peripheral nerve block, RA = regional anesthesia (PNB or EA).

Data are presented as n (%) unless stated otherwise.

**Figure 2.**
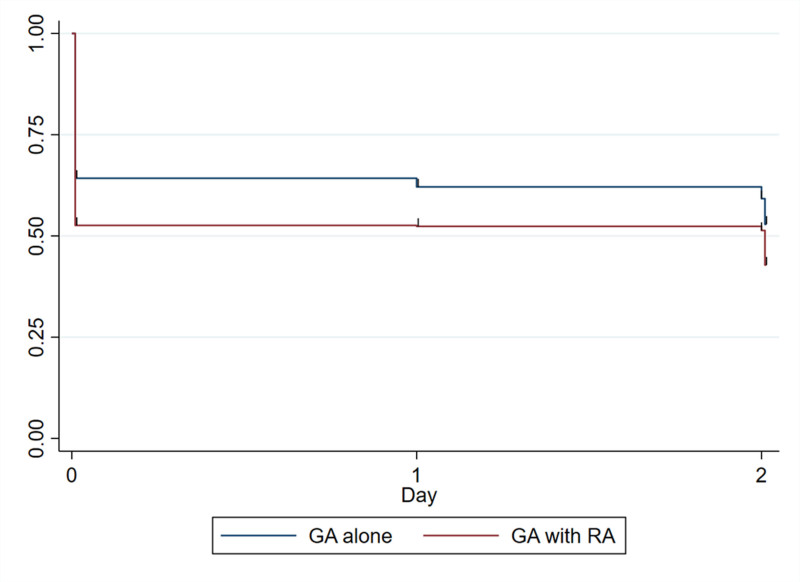
Stabilized inverse probability of treatment weighting Kaplan–Meier analysis of the incidence of postoperative nausea and vomiting. GA = general anesthesia, RA = regional anesthesia (peripheral nerve block or epidural anesthesia).

Figure 2 shows the results of the sIPTW Kaplan–Meier analysis. Overall, after sIPTW, the incidence of PONV was 57.2% in the GA–RA group and 47.1% in the GA-alone group.

The sIPTW univariable Cox regression analysis revealed a slight difference in the overall incidence of PONV (HR, 1.25; 95% CI, 1.24 to 1.26; *P* < .001) between the GA–RA and GA-alone groups. Table 2 shows the HRs for PONV incidence that were determined using the sIPTW multivariable Cox regression analysis. Patients were higher odds RA (HR, 1.20; 95% CI, 1.19 to 1.21; *P* < .001), female sex (HR, 1.67; 95% CI, 1.65 to 1.68; *P* < .001), inhaled anesthesia (HR, 1.09; 95% CI, 1.08 to 1.10; *P* < .001), intraoperative or postoperative opioid usage (HR, 1.69; 95% CI, 1.65 to 1.73; *P* < .001), laparoscopic surgery (HR, 1.05; 95% CI, 1.04 to 1.06; *P* < .001), and lower age (HR, 0.83; 95% CI, 0.82 to 0.83; *P* < .001). Proportional hazards are presented in Figure S1 and 2, Supplemental Digital Content, https://links.lww.com/MD/P215.

**Table 2:** Cox regression proportional HRs: multivariable model after stabilized inverse probability of treatment weighting.

	HRs	95% CIs	
RA	1.2	1.19–1.21	*P* < .001
Female	1.67	1.65–1.68	*P* < .001
Anesthesia duration (≥60 min)	1.99	1.95–2.04	*P* < .001
Age (≥50 yr-old)	0.83	0.82–0.83	*P* < .001
Smoking	1	1.00–1.01	*P* = .279
Inhaled anesthesia	1.09	1.08–1.10	*P* < .001
Intra, postoperative opioid	1.69	1.65–1.73	*P* < .001
laparoscopic surgery	1.05	1.04–1.06	*P* < .001

CIs =  confidence intervals, HR = hazard ratios, RA = regional anesthesia.

The E-value indicated that a confounder would have to be associated with the exposure and PONV by a value of 1.53 each on a relative risk scale to negate the observed association between GA with RA and PONV. Similarly, the CI for the observed association with PONV could be moved to include the null by an unmeasured confounder associated with exposure and PONV by a factor of 1.51.

**Figure 3.**
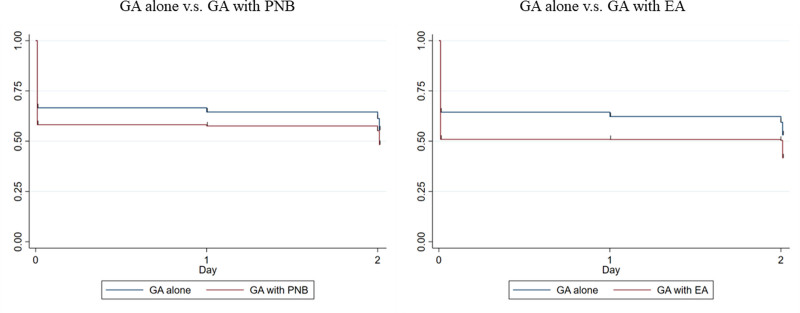
Subgroup analysis. GA = general anesthesia, PNB = peripheral nerve block, EA = epidural anesthesia.

The results of the subgroup analyses are presented in Figure 3. The sIPTW multivariable Cox regression analysis revealed a slight difference in the overall PONV incidence (HR, 1.15; 95% CI, 1.14 to 1.16; *P* < .001) between the GA–PNB (n = 54,792) and GA-alone groups. The analysis also revealed a difference in the overall PONV incidence (HR, 1.24; 95% CI, 1.23 to 1.25; *P* < .001) between the GA–EA (n = 194,641) and GA-alone groups. GA–PNB and GA–EA were associated with PONV incidence after abdominal surgery.

The sIPTW RMST analysis revealed an RSMT of 1.05 (95% CI, 1.05 to 1.06) days in the GA–RA group and 1.27 (95% CI, 1.26 to 1.27) days in the GA-alone group. The difference in the mean survival time was 0.21 days (95% CI, 0.21 to 0.22; *P* < .001). This indicates that the PONV incidence was higher in the GA–RA group near the date of surgery.

The sensitivity analysis using multiple imputation yielded results consistent with those from the original dataset, suggesting that missing data had minimal impact on the primary estimates. Missing values were minimal for age (0.7%), sex (0.7%), BMI (1.8%), emergency (0.7%).

For the overall incidence of PONV, the HRs derived from the imputed datasets were 1.25 (95% CI, 1.24 to 1.26; *P* < .001) for the univariable model and 1.20 (95% CI, 1.19 to 1.21; *P* < .001) for the multivariable model, respectively, for GA–RA and GA alone. This similarity suggests that the findings were robust to the imputation process, supporting the validity of the primary analysis results.

## 4. Discussion

In this study, we used a national inpatient database in Japan to examine the effect of RA (PNB or EA) under GA on the incidence of PONV in patients undergoing abdominal surgery. Unlike common beliefs, RA under GA was associated with a slight increase in the PONV incidence in the present study. Sensitivity analyses confirmed the robustness of our findings. RA (PNB or EA) is generally believed to decrease PONV by reducing postoperative pain. However, this does not seem to hold true in Japan.

Following an analysis of 22 trials including 1154 participants, Cochrane Database stated that the incidence of vomiting within 24 hours did not differ with EA (risk ratio 0.84, 95% CI 0.57 to 1.23; low quality of evidence).^[8]^ The current guidelines^[7]^ provide only a vague recommendation for the “avoidance of GA by the use of RA.” However, it is difficult to demonstrate PONV suppression following EA under GA due to various risk factors, such as the use of volatile agents, systemic intraoperative opioids, age, sex, anesthesia duration and laparoscopic surgery. Nevertheless, we have been able to obtain more generalized results using large data sets.

Some studies have demonstrated a relationship between PNB and PONV. However, the small sample sizes and variability in the techniques used in the previous studies make this problem difficult to solve. Moreover, only few studies have investigated the relationship between PONV and PNB with GA as the primary endpoint; this has been considered a secondary endpoint in most studies. Furthermore, few studies have observed changes in PONV over time. Whether PNB is associated with PONV suppression remains controversial, although this is a secondary issue. Following a review of a small number of studies published to date, Charlton et al reported a lack of an apparent reduction in PONV with the use of a transversus abdominis plane (TAP) block or sedation.^[15]^ Kitlik et al evaluated the effects of a TAP block on postoperative analgesia and opioid consumption in living liver donors in whom a right “J” abdominal incision was made. They indicated that the TAP block reduced the 24-hour postoperative morphine consumption and contributed to analgesia but did not reduce PONV.^[16]^ Sultan et al compared TAP block with wound infiltration for postoperative analgesia following cesarean delivery; no significant differences were observed in PONV.^[17]^

In principle, pain suppression by RA may be strongly influenced by intraoperative and postoperative opioids. Mauermann et al reported that intraoperative opioid use was the most significant risk factor for postoperative PONV.^[18]^ Visceral pain is difficult to relieve with PNB; therefore, at least one opioid must be administered during abdominal surgery. A byproduct of this seems to be PONV development. Whether PNB suppresses PONV, which appears to be closely related to opioids, remains controversial.

A key strength of this study was its large sample size of patients undergoing abdominal surgery. Extensive and versatile data increase the generalizability of the study findings. Our database also allowed for a Cox proportional hazards analysis weighted by sIPTW. This model was successful because a multivariate model with existing risk factor inputs was previously reported.^[6,7,19]^ This increased the robustness of the analysis. The RMST is also a strength. The sensitivity analyses further increased the robustness. The primary outcome is more reliable because it was identified by the therapeutic agent rather than by the name of the disease. Unlike in countries where patients are discharged from the hospital immediately after surgery, in Japan, patients remain in the hospital ward for an extended period after surgery. Drugs for PONV are administered as per the patient’s request; thus, the relevant data are highly reliable.

### 4.1. Limitation

This study had several limitations. First, it was a retrospective observational study; therefore, unmeasured confounders cannot be ruled out. However, we determined an E-value of 1.53. It is unlikely that the influential factors will exceed this value. Second, we could not collect data on the “history of PONV” and “motion sickness” (known risk factors for PONV) and on what PNBs were performed, as well as opioid consumption and route of administration, because these data are not included in the DPC database. It may be possible in the future to collect electronic medical records tied to medical claims data. Third, unlike in other countries, 5HT3 receptor antagonists (such as ondansetron or granisetron) were not administered during the study period. If 5HT3 receptor antagonists were administered prophylactically, as is the case now, the results might have been different. However, the present study examined the association between PONV and RA without interference from 5HT3 receptor antagonists and without confounding factors. Based on results of this study, we recommend prophylactic administration of 5HT3 receptor antagonists during abdominal surgery. Fourth, our data were almost exclusively from the Japanese. Whether race or ethnicity is a risk factor for PONV remains controversial. The incidence of PONV appears to be lower in Asians than in Western countries.^[20]^ Fifth, this study involved a complete case analysis; therefore, cases with missing values were excluded. However, because these cases accounted for only approximately 1.9% of the total cases.

## 5. Conclusion

In this large-scale retrospective analysis of patients undergoing abdominal surgery, the combination of GA and RA was associated with a higher incidence of PONV compared to GA alone. These findings challenge the widely held assumption that RA confers a protective effect against PONV. Although our results are derived from a specific surgical population, they underscore the need to critically reassess the role of RA in PONV prevention across various clinical contexts. Future prospective studies are warranted to validate these observations, explore underlying mechanisms, and inform evidence-based anesthesia strategies aimed at mitigating PONV risk.

## Acknowledgments

The authors would like to thank Editage (www.editage.com) for English language editing.

## Author contributions

**Conceptualization:** Manabu Yoshimura.

**Data curation:** Manabu Yoshimura, Hiroko Shiramoto.

**Formal analysis:** Manabu Yoshimura.

**Funding acquisition:** Manabu Yoshimura.

**Investigation:** Manabu Yoshimura.

**Methodology:** Manabu Yoshimura, Takaaki Tanemoto.

**Project administration:** Manabu Yoshimura.

**Resources:** Manabu Yoshimura.

**Software:** Manabu Yoshimura, Takaaki Tanemoto.

**Supervision:** Takaaki Tanemoto, Hiroko Shiramoto, Mami Koga, Yasuhiro Morimoto.

**Validation:** Manabu Yoshimura.

**Visualization:** Manabu Yoshimura.

**Writing – original draft:** Manabu Yoshimura.

**Writing – review & editing:** Takaaki Tanemoto, Hiroko Shiramoto, Mami Koga, Yasuhiro Morimoto.

## Supplementary Material


