# Supplementary material for: Prevalence of antiemetic administration after abdominal surgery with or without a regional anesthesia under general anesthesia in a nation-wide population-based study
Source: Medicine (Baltimore). 2025 Aug 22;104(34):e42894. doi: 10.1097/MD.0000000000042894 (PMC12385035; doi:10.1097/MD.0000000000042894)
Supplement: Supplementary file 1 [file medi-104-e42894-s001.pdf]

Supplement Table 1. The definition of difficulty in anesthesia induction

This definition is defined by the Ministry of Health, Labour and Welfare in Japan.

Patients who are difficult to anaesthetize are those listed below, and shall be evaluated according to their condition before anesthesia.

(a) Patients with heart failure (limited to those with NYHA degree III or higher)

(b) Patients with angina pectoris (limited to those with CCS classification III or higher)

(c) Patients with myocardial infarction (limited to those within 3 months of onset)

(d) Patients with aortic regurgitation, mitral regurgitation, or tricuspid regurgitation (limited to those with moderate or severe regurgitation)

(e) Aortic stenosis (limited to aortic valve stenosis with a trans-aortic blood flow velocity of 4 m/sec or more, aortic valve mean pressure gradient of 40 mmHg or more, or aortic valve opening area of 1.0 cm^2 or less) or mitral stenosis (limited to those with a mitral valve opening area of 1.5 cm^2 or less)

(f) Patients with an implantable pacemaker or an implantable defibrillator

(g) Patients with congenital heart disease (limited to those with a mean pulmonary artery pressure of 25 mmHg or more as determined by cardiac catheterization or those with equivalent pulmonary hypertension as determined by echocardiography)

(h) Patients with pulmonary arterial hypertension (limited to those with mean pulmonary arterial pressure of 25 mmHg or more according to cardiac catheterization, or those with equivalent pulmonary hypertension diagnosed by cardiac ultrasonography)

(i)Respiratory failure (limited to those with an arterial blood oxygen partial pressure of less than 60 mmHg or an arterial blood oxygen partial pressure/inhaled air oxygen fraction ratio of less than 300)

(j) Patients with ventilation impairment (limited to those with a 1-second rate of less than 70% and a lung capacity ratio of less than 70%)

(k) Patients with bronchial asthma (limited to those with recurrent attacks of a middle attack or greater despite treatment)

(l) Patients with diabetes mellitus (limited to those with HbA1c of 8.0% or more for JDS (8.4% or more for NGSP), fasting blood glucose of 160 mg/dL or more, or 2-hour postprandial blood glucose of 220 mg/dL or more)

(m) patients with renal failure (limited to those with a serum creatinine level of 4.0 mg/dL or higher)

(n) Patients with liver failure (limited to those with Child-Pugh classification B or higher)

(o) Patients with anemia (limited to those with Hb less than 6.0g/dL)

(p) Patients with impaired blood coagulation (limited to those with a PT-INR of 2.0 or higher)

(q) Patients with DIC

(r) Patients with thrombocytopenia (limited to those with platelets of less than 50,000/uL)

(s) Patients with sepsis (limited to those with SIRS)

(t)Patients in shock (limited to those with a systolic blood pressure of less than 90 mmHg)

(u) Patients with complete spinal cord injury (limited to those higher than the fifth thoracic vertebra)

(v) Patients on cardiopulmonary support

(w)Patients undergoing dialysis

(x) Patients undergoing intra-aortic balloon pumping

(y) Patients with a BMI of 35 or more
