# Supplementary material for: Prevalence of antiemetic administration after abdominal surgery with or without a regional anesthesia under general anesthesia in a nation-wide population-based study
Source: Medicine (Baltimore). 2025 Aug 22;104(34):e42894. doi: 10.1097/MD.0000000000042894 (PMC12385035; doi:10.1097/MD.0000000000042894)

Supplementary figure 1. Schoenfeld residuals test

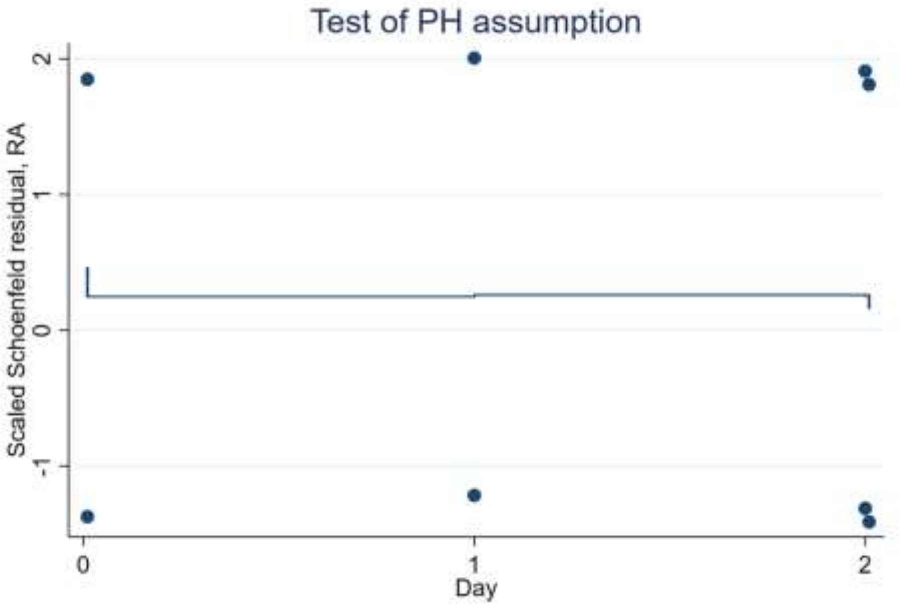

Supplementary figure 2. Complementary log plots

GA, general anesthesia; RA, regional anesthesia (peripheral nerve block or epidural anesthesia)

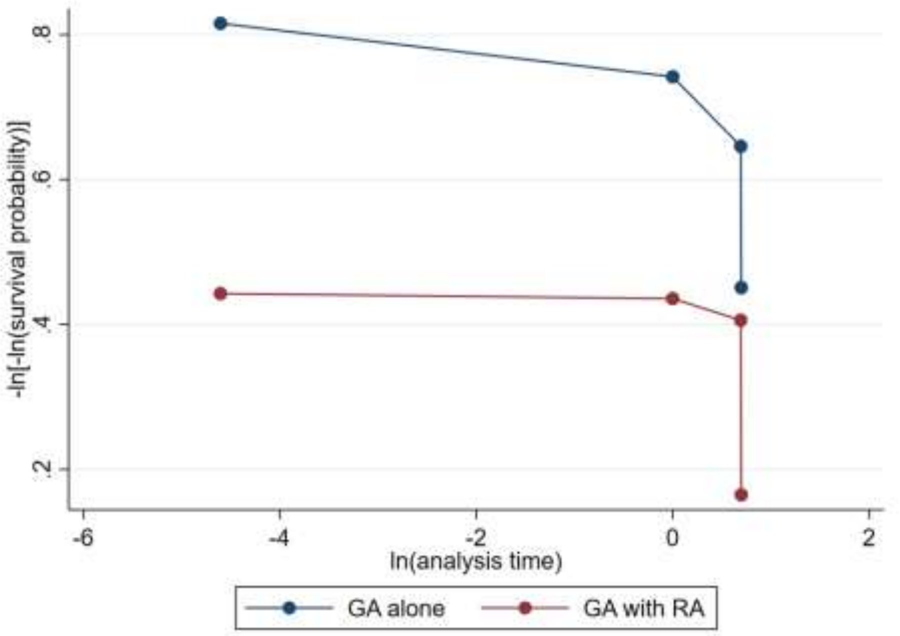

Supplement: Supplementary file 2 [file medi-104-e42894-s002.pdf]
